# Using Accelerometers to Measure Physical Activity in Older Patients Admitted to Hospital

**DOI:** 10.1155/2018/3280240

**Published:** 2018-10-18

**Authors:** Peter Hartley, Victoria L. Keevil, Kate Westgate, Tom White, Søren Brage, Roman Romero-Ortuno, Christi Deaton

**Affiliations:** ^1^Department of Public Health and Primary Care, University of Cambridge, CB2 0SR, UK; ^2^Department of Physiotherapy, Cambridge University Hospital NHS Foundation Trust, CB2 0QQ, UK; ^3^Department of Medicine for the Elderly, Cambridge University Hospital NHS Foundation Trust, CB2 0QQ, UK; ^4^MRC Epidemiology Unit, University of Cambridge, CB2 0SL, UK; ^5^Trinity College Dublin, Discipline of Medical Gerontology, Mercer's Institute for Successful Ageing, St James's Hospital, Dublin 8, Ireland

## Abstract

**Background:**

Low levels of physical activity in older patients during hospitalization have been linked to loss of functional ability. Practical methods of measuring physical activity are needed to better understand this association and to measure the efficacy of interventions. The aims of this study were to evaluate the feasibility of using accelerometers to discriminate between lying, sitting, standing, and standing and moving and to determine the acceptability of the method from the patients' perspective.

**Methods:**

A convenience sample of 24 inpatients was recruited. Participants wore accelerometers on their thigh and on their lower leg (just above the ankle) for 48 hours during their hospitalization. Postural changes and movement during the 48 hours were differentiated using derived pitch angles of the lower leg and thigh, and nongravity vector magnitude of the lower leg, respectively.

**Results:**

On average, patients were lying for 61.2% of the recording time, sitting for 35.6%, standing but not moving 2.1%, and standing and moving 1.1%. All participants found the accelerometers acceptable to wear.

**Conclusions:**

The methodology described in this study can be used to differentiate between lying, sitting, standing, and moving and is acceptable from a hospitalized older person's perspective.

## 1. Background

Low levels of physical activity in older hospitalized adults are well documented [1–4] and have been linked with declines in functional ability [5] and muscle strength [6]. As such, there is a need to measure physical activity when investigating hospital-associated functional decline in older patients. Accelerometers are tools used to objectively measure levels of activity in research and have been used extensively in community-dwelling adults [7, 8].

Accelerometers have been worn on a variety of anatomical positions, but most often worn on the hip or wrist, with the latter being the most common due to its high acceptability to study participants [7–9]. In community cohorts and epidemiological studies, accelerometer data are usually reported in terms of time spent at different intensities of physical activity [10]. In hospital cohorts, this information is somewhat redundant due to the severity of inactivity and low intensity of all activity. Typically, research using accelerometer-measured activity in hospital has used step count or time spent upright (standing or walking versus sitting or lying) to quantify activity [11]. However, it would be desirable to measure hospital activity in terms of time spent in specific postures (e.g., lying, sitting, and standing) and time spent walking [12–14]. This is because a more nuanced characterization of mobility patterns in older hospitalized patients may facilitate the design of specific physical interventions to try to combat hospital-associated functional decline. For instance, it has been suggested that sitting in a chair may have a therapeutic benefit in some patients [15, 16], but prolonged sitting compared to resting lying on a bed may also be detrimental [17]. To accurately differentiate between lying in bed, sitting in a chair, and standing, two sensors are required which can be positioned either on the thigh and lower leg [13, 14] or on the sternum and thigh [12].

To our knowledge, six studies have validated methods for using two accelerometers in hospitalized patients for specifically measuring posture [12–14, 18–20], including four in an acute ward setting [13, 14, 18, 20]. Four of the six studies placed accelerometers on the thigh and lower leg [13, 14, 18, 19] and two on the thigh and sternum [12, 20]. Four of these studies specified the accelerometer model used: one study used the ADXL202 [12], one used the ActivPAL [20], and two used the PAL2 device [18, 19]. Two studies reported their algorithms for defining lying, sitting, and standing positions in relation to the detected angles of the accelerometers [12, 14]. Pedersen* et al*. [14] attached sensors to the participants' thigh and lower leg, and Culhane* et al*. [12] attached sensors to the thigh and sternum. Both reported high levels of accuracy in all three positions. Two studies had a fourth category of “movement” [12, 20], one using the ActivPAL and one using ADXL202 accelerometers; Culhane* et al*. [12] reported high rates of movement detection accuracy, and Taraldsen* et al*. [20] reported low level of step count accuracy when walking speed was below 0.47 m/s.

The aims of this feasibility study were to develop a method to discriminate between lying, sitting, standing, and moving (upright), in older hospitalized patients using two accelerometers (on the thigh and lower leg), and to determine the feasibility and acceptability of the method from the patients' perspective.

## 2. Methods

### 2.1. Study Design

This is a feasibility study using a cross-sectional single center cohort design. Ethical approval was granted by the South Central Hampshire A Research Ethics Committee (reference 17/SC/0219).

### 2.2. Setting

The study was conducted on inpatient wards of the Department of Medicine for the Elderly (DME) at Cambridge University Hospitals (CUH) NHS Foundation Trust, England, United Kingdom. CUH is a tertiary university teaching hospital in the English National Health Service (NHS) with approximately 1000 inpatient beds. DME wards have approximately 150 beds and specialize in the provision of Consultant (i.e., senior physician)-led multidisciplinary Comprehensive Geriatric Assessment for older adults admitted to the hospital. The clinical characteristics of this setting have been described elsewhere [21, 22].

### 2.3. Study Period

Patients were recruited between May and October 2017.

### 2.4. Population

Convenience sample of patients admitted to DME wards over the age of 70, under the care of a DME Consultant (Senior Attending Physician), with an expected hospital stay of more than 72 hours. Our Patient and Public Involvement (PPI) group suggested that men and women might have different views regarding acceptability of wearing accelerometers. We therefore recruited 12 men and 12 women, using the guidance of the National Institute for Health Research (NIHR) of recruiting 12 participants per group in a feasibility study [23].

*Exclusion criteria *are as follows: inability to provide informed consent as assessed by the researcher taking consent; if the patient was receiving end of life care; disagreement to participation by the DME Consultant in charge of the patient's care; if the clinical team had any concerns regarding skin integrity around proposed accelerometer sites; allergy to adhesive dressings; or if the patient was already actively involved in another research study.

### 2.5. Procedures

Potential participants were identified by members of the clinical team who sought permission from the individuals for them to be approached by a member of the research team. If permission was given, a member of the research team (PH, a Physiotherapist with 8 years' clinical experience) would discuss the study with the participant and provide them with further written information regarding the study. Written information was produced in conjunction with the PPI group prior to the study. Participants were given 24 hours to consider their participation before being asked to provide written consent. Mental capacity was assessed at both meetings by PH and confirmed verbally with the DME Consultant.

**Figure 1:**
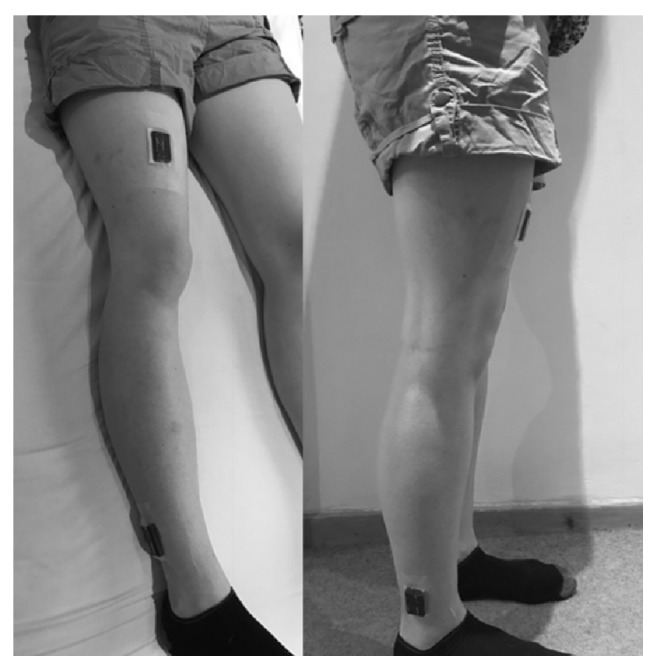
Placement of accelerometers; the accelerometers are orientated with the micro-USB port towards the feet.

After providing written consent, participants were fitted with two accelerometers (AX3, Axivity, Newcastle upon Tyne, UK) on the same leg, the first on the front of the thigh, proximal to the patella (a third of the way between the knee and the hip). The second sensor was placed on the side of the lower leg approximately 5cm above the lateral malleolus of the ankle (Figure 1). The lateral side of the lower leg was chosen as opposed to the anterior to minimize the risk of excessive pressure on the skin if the participants were to lie in bed with their legs crossed. The research team considered the potential risk of increased pressure on the skin if the participants were to lie on their side. However due to the pressure relieving mattresses the hospital uses, the risk was felt to be sufficiently low to proceed. In addition, the accelerometer sites were checked daily for comfort and skin integrity. The accelerometers were placed on the right leg unless there were clinical reasons prohibiting its placement (e.g., skin lesion) or the participant had a strong preference for the left leg. The thigh and lower leg sensor locations were preferred to using the thigh and sternum locations by both our PPI group and members of the clinical teams. The preference of location was based on both practical reasons (e.g., potential interference with routine clinical procedures) and the expected acceptability to patients. Following advice from our PPI group and clinical teams, the accelerometers were placed over a piece of gauze to avoid direct friction with the skin and fixed to the skin with a 10cm by 12cm transparent dressing (3M Tegaderm Film). The AX3 can be submerged in water at a 1.5m depth for 1 hour and therefore there were no restrictions on participants being able to shower or wash. The device has been used in the UK Biobank study where it was worn on the wrist by over 100,000 participants [9] and has demonstrated equivalent signal vector magnitude output on multiaxis shaking tests to the GENEActiv accelerometer [24].

Participants were asked to wear the sensors continuously over a 48-hour period. Patients were instructed to inform clinical staff if they experienced any discomfort at the accelerometer sites, in order to have them immediately removed. A member of the research team checked skin integrity (through the transparent dressing) on a daily basis. Following the 48-hour monitoring period, a member of the research team removed the accelerometers and participants were asked to complete a questionnaire regarding the acceptability of their monitoring experience (Box 1). A 48-hour wear time was felt to be a sufficient period of time to assess acceptability by both the research team and PPI group. If a patient's discharge date was changed to within the 48-hour period after study commencement, or if he/she needed to undergo an MRI scan during the monitoring period, the accelerometers were removed and the questionnaire was completed at that point.

### 2.6. Other Measurements

For descriptive purposes, we recorded the participants' age, sex, comorbidity burden, acute illness severity, degree of frailty, level of mobility, reason for hospital admission, and length of hospital stay prior to participation in the study.

Comorbidity burden was measured using the Charlson Comorbidity Index (CCI), which is based on patients' diagnoses as coded by the World Health Organization's International Classification of Diseases (10^th^ version) [25]. Acute illness severity was measured by the National Early Warning Score [26], recorded at the points of study entry and exit.

Frailty was measured using the Clinical Frailty Scale (CFS). The scoring of the CFS is based on a global assessment of patients' comorbidity symptoms, cognition, and their level of physical activity and dependency on activities of daily living [27]. The possible scores are 1 (very fit), 2 (well), 3 (managing well), 4 (vulnerable), 5 (mildly frail), 6 (moderately frail), 7 (severely frail), 8 (very severely frail), and 9 (terminally ill). The Modified Functional Ambulatory Category (MFAC) was used to describe the participants' level of functional mobility. The MFAC is a seven-point scale with the following scores: 1: cannot ambulate and requires manual assistance to sit; 2: is able to sit for a minute without support; 3: requires continuous manual assistance of 1 person to support body weight in order to ambulate; 4: requires continuous or intermittent light manual assistance of 1 person to maintain balance when ambulating; 5: requires supervision for safety reasons when ambulating; 6: is able to ambulate independently on level surfaces; 7: is able to ambulate independently on level and nonlevel surfaces [28]. As mobility was only assessed in the hospital ward (on a level surface), for the purposes of this study participants could score a maximum of 6. Members of the direct clinical care teams described each participant's functional mobility at the beginning and end of their participation in the study to provide an MFAC score.

Acceptability was assessed by a specifically designed questionnaire (Box 1); other acceptability outcomes were as to whether the sensors had been worn for the intended time period; or if any concerns had been raised by participants, their families, or members of staff regarding skin integrity or bruising around the sensor sites.

### 2.7. Accelerometer Data Acquisition

The accelerometers were set up to record movement with a sampling frequency of 100 Hertz and a range of ± 4g using OMGUI software developed by Newcastle University [29]. Following removal of the devices, the same software package was used to download the recorded data.

### 2.8. Accelerometer Data Processing

Data processing was performed using pampro [30], an open source software package (https://github.com/Thomite/pampro). The raw acceleration data was autocalibrated to local gravity using the method described by van Hees* et al*. [31]. Due to the placement of the devices and the relatively short duration of measurement, calibration factors were calculated based on all data collected by each device, rather than within a single record. Machine noise was filtered out by applying a low-pass filter at 20 Hertz.

To define the participants' position or activity, three variables from the accelerometers were derived: Euclidean Norm Minus One (ENMO), thigh pitch (elevation) angle: and lower leg pitch angle. ENMO was calculated using the following formula: $ENMO=\left(\sqrt{x^{2}+y^{2}+z^{2}}\right)-1$, where x, y and z refer to the acceleration in g detected along the x, y, and z axes, respectively. ENMO subtracts 1g from the Euclidian norm (vector magnitude) and truncates negative values to zero at sample level to remove the gravity component from the signal, thereby isolating activity-related acceleration [32]. It is normal practice to use nonwear detection procedures to identify when the accelerometers have been removed [33]. As only the research team fitted or removed the accelerometers, compliance with wear was known and therefore nonwear detection was not required.

When the accelerometer is still, the pitch angle along its primary axis x is calculated using the following formula: $\tan^{-1}\left(x/\sqrt{y^{2}+z^{2})}\right)\left(180.0/\pi \right)$. The formula provides angles from +90° to -90°, where +90° or -90° represent the accelerometer's x-axis being aligned parallel to the earth's gravity vector (straight up or straight down, respectively), and 0° represents the accelerometer's x-axis angle being aligned perpendicular to the earth's gravity vector. Using our orientation of the accelerometers (micro-USB port towards the feet), 90° represented the patient being “upright” or “standing”.

**Figure 2:**
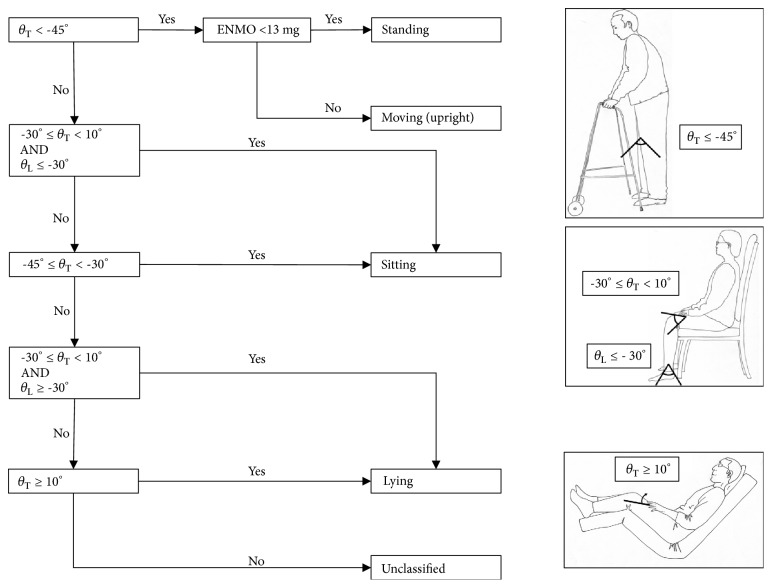
Algorithm for determining participant posture and movement (*θ*_T_ = pitch angle of thigh accelerometer, *θ*_L_ = pitch angle of lower leg accelerometer, and ENMO from lower leg accelerometer).

Each of the three signals was summarized from sample-level data into 5-second epochs. Before further variables were derived, a correction was made for the lower leg accelerometer pitch angle. When the accelerometer was attached to the patient, the researchers aimed to align the accelerometer with the lower leg, so that when the lower leg was perpendicular to the floor, the pitch angle would be -90°. Due to the practical constraints of positioning a patient when fitting the accelerometer, this was not always accurate. As all included participants were known to have walked during the study period it was assumed that the minimum lower leg pitch angle was -90° in all participants. All lower leg pitch recordings for each participant were therefore reduced by the difference between their minimum recorded pitch angle and -90°. Every 5-second window was classified as one of: “lying”, “sitting”, “standing”, and “moving (upright)”, based on the rules given in Figure 2. The static positions were based on the algorithm validated by Pedersen* et al*. [14]. The levels of correspondence between inferred position from accelerometer data and observed positions for lying, sitting, and standing were reported by Pedersen* et al*. [14] as 90.8–100%, 95.3–98.6%, and 89.6–96.5%, respectively. ENMO values were used from the lower leg accelerometer to define whether or not a person was moving (when already determined to be standing according to the positional criteria). Our previous work has determined usual walking speeds in this population at the point of hospital discharge as 0.33 m/s (IQR 0.21–0.50 m/s), a very low speed compared to those reported in community based samples [34]. A cut-off of ENMO value of >13 milligravity units (mg) from the accelerometer on the lower leg was used to define a participant as “moving”. This value has previously been used with wrist accelerometers to define stationary periods [9, 35]. We tested the threshold value using data from accelerometers worn by members of the research team on their lower leg when stationary, and when walking on a treadmill at 0.1 m/s. Stationary and walking at 0.1 m/s, median (IQR) ENMO values were 3.7mg (1.7–10.5mg) and 16.4mg (13.9–20.1mg), respectively.

### 2.9. Data Analysis

Statistics were presented as mean and standard deviation, or as a count and percentage for categorical variables. For continuous variables with a nonnormal distribution we reported median and interquartile range (IQR) or median and range if the number of data points was less than 25.

## 3. Results

Participant characteristics are described in Table 1. Female participants had a median age of 81.5 years and male participants of 79.5 years. The median MFAC score when the accelerometers were first attached to the participants was 5 (requiring supervision to ambulate for safety purposes), although this improved to 6 (able to walk independently on level surfaces) by the end of the measurement period. Other clinical characteristics and outcomes are summarized in Table 1.

**Figure 3:**
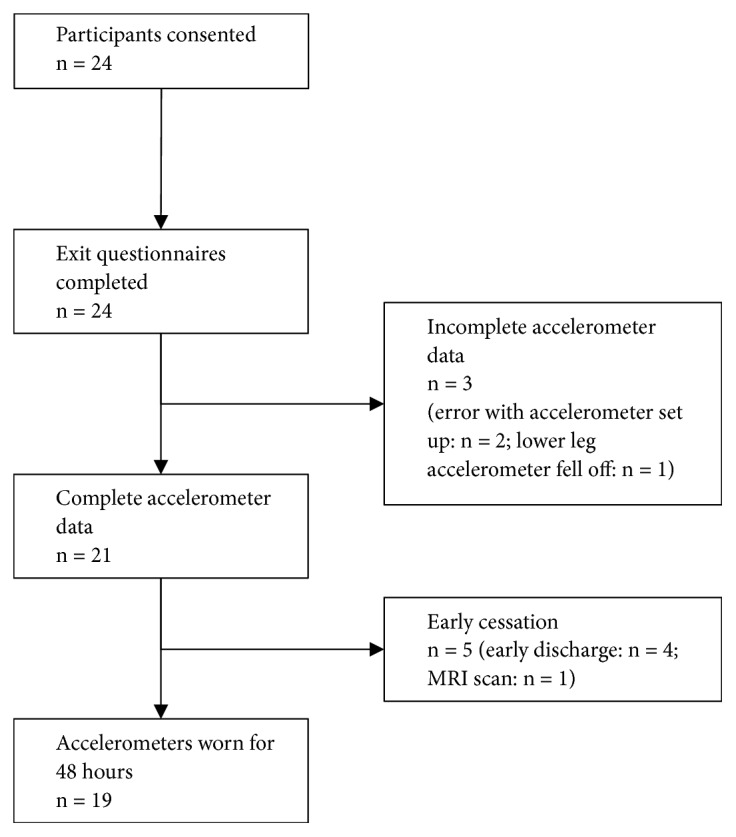
Flow of participants through study.

Three participants' activity data was not captured; set-up errors were made for two participants, on one or both of the accelerometers, and in one case the lower leg accelerometer fell off and the participant declined to have it refitted as they were concerned that it could get lost. In five further cases, the accelerometers were removed prematurely due to earlier than expected discharges or MRI scans, but in all those cases at least 24 hours of data was collected (Figure 3).

All participants completed the acceptability questionnaire. All participants reported ‘no discomfort', with 16 participants reporting similar comments to the effect of having forgotten that they were wearing the accelerometers. All participants reported that their sleep had not been affected by wearing the accelerometers. All but one participant reported that they would wear the accelerometers again in a future study.

**Figure 4:**
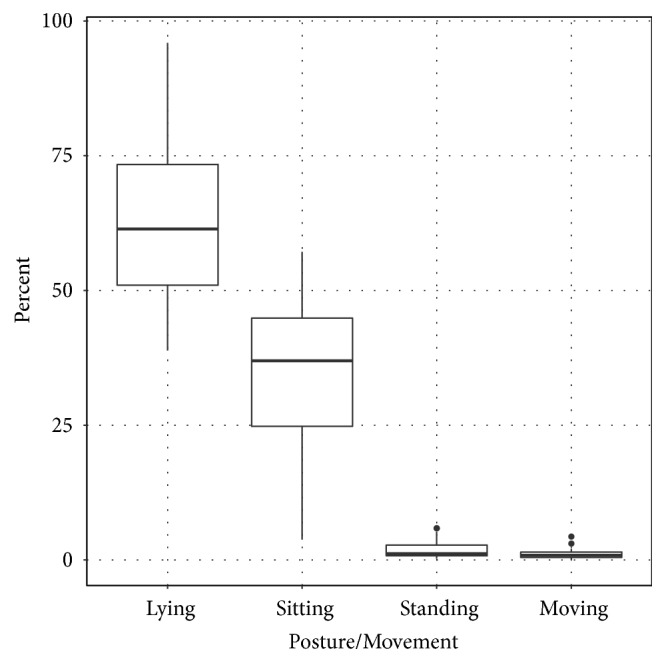
Summary of posture and movement during measurement period.

**Figure 5:**
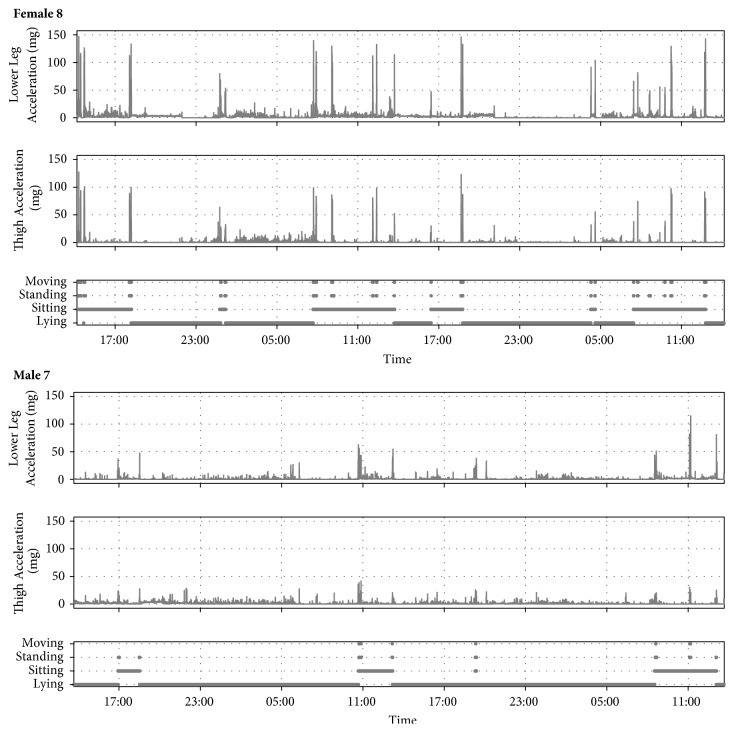
Examples of participant's activity during study period.

The levels of activity for each participant are summarized in Table 1 and Figure 4. On average, patients were lying for 62.6% (±16.3) of the recording time, sitting for 34.2% (±14.7), standing but not moving 2.0% (±1.9), and standing and moving 1.2% (± 1.1). Examples of participant activity over the study period are illustrated in Figure 5.

## 4. Discussion

This study described a novel method of measuring modalities of physical activity in older patients during an acute hospitalization. The method was feasible and allowed differentiation between lying, sitting, standing, and moving (upright). Furthermore, this measurement was considered to be acceptable from the patients' perspective.

Our study built upon previous work using 2 accelerometers to differentiate positions of hospitalized patients. We believe this is the first study to use accelerometers on the thigh and lower leg to differentiate between lying, sitting, standing, and moving in acutely hospitalized patients.

Compared to the PAL2 device, the AX3 device and the methods we used to attach it to the participants may be more acceptable. Using the PAL2 device, Kramer* et al*. [18] reported two of the eight participants reported the device being uncomfortable.

The amount of time spent standing or walking was similar to that recorded by other studies of similar populations, though the amount of time spent lying was lower. Pedersen* et al*. [14] reported that their ambulatory patients were lying 70% of the time, sitting 21%, and standing or walking 4%; Brown* et al.* [1] reported participants lying 83% of the time, sitting 12.9%, and standing or walking 3.8%.

We used a convenience sample, and the duration of the study was a maximum of 48 hours; it is possible that, with a larger random sample and longer study duration, the reported acceptability of the devices may have been different. Furthermore, our participants had on average been in hospital 4 days prior to starting the study; it is possible that they would have found the accelerometers less acceptable in the first days of hospitalization. The movement threshold between standing and moving has not been validated in the patient population and is likely to be inaccurate at walking speeds of less than 0.1 m/s. This is relevant given the known slow walking speed of our population; however the significance of standing versus walking at such a slow speed is not known.

## 5. Conclusion

The methodology described in this study can be used to differentiate between lying, sitting, standing, and standing and moving and is very acceptable from a hospitalized patient's perspective. Work is now required to examine the significance of the derived variables on hospital outcomes.

## Figures and Tables

**Box 1:**
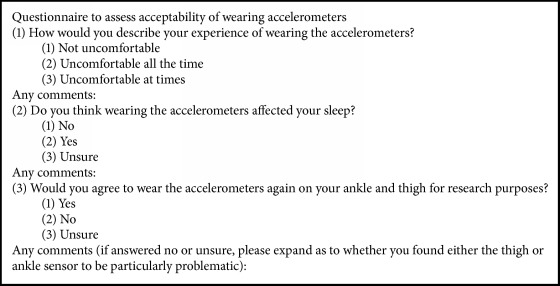
Acceptability questionnaire.

**Table 1:** Participant characteristics.

	**All **	**Female **	**Male **
**Clinical characterization**	**(n = 24)**	**(n = 12)**	**(n = 12)**

**Age (years) **	80.5 (70.0–95.0)	81.5 (71.0–89.0)	79.5 (70.0–95.0)
**Days since admission**	4 (3–7)	5 (3–8)	3 (3–5)
**CCI**	1.5 (0–9)	1 (0–6)	2 (0–9)
**CFS**	5 (1–7)	4 (1–7)	6 (2–7)
**MFAC Start**	5.5 (2–6)	5.5 (2–6)	5 (2–6)
**MFAC End**	6 (2–6)	6 (2–6)	6 (2–6)
**NEWS Start**	1.5 (0–6)	1 (0–4)	2.5 (0–6)
**NEWS End**	1 (0–5)	1 (0–4)	1.5 (0–5)

**Accelerometer characterization**	**n = 21**	**n = 9**	**n = 12**

**Average ENMO lower leg (mg)**	2.1 (±11.2)	2.6 (±12.2)	1.9 (±10.6)
**Time spent lying (**%**)**	62.6 (±16.3)	54.4 (±10.0)	68.7 (±17.7)
**Time spent sitting (**%**)**	34.2 (±14.7)	41.8 (±8.9)	28.5 (±15.9)
**Time spent standing (**%**)**	2.0 (±1.9)	2.5 (±2.4)	1.7 (±1.5)
**Time spent moving (upright) (**%**)**	1.2 (±1.1)	1.3 (±0.9)	1.1 (±1.2)

Data presented as median (range) or mean(±SD).

CCI = Charlson Comorbidity Index, CFS = Clinical Frailty Scale, MFAC = Modified Functional Ambulatory Category, NEWS = National Early Warning Score, and ENMO = Euclidean Norm Minus One.

## Data Availability

The data used to support the findings of this study have not been made available as this was not part of our ethics application in order to protect patient confidentiality.
